# Supplementary material for: Drivers of soil microbial and detritivore activity across global grasslands
Source: Commun Biol. 2023 Dec 1;6:1220. doi: 10.1038/s42003-023-05607-2 (PMC10692199; doi:10.1038/s42003-023-05607-2)
Supplement: Supplementary file 2 — Supplementary File.pdf [file 42003_2023_5607_MOESM2_ESM.pdf]

Supplementary information

Table S1: Included sites with information on continent, country, grassland type, start of treatment, elevation, latitude (in °), longitude (in °), mean annual temperature (MAT, in °C), and mean annual precipitation (MAP, in mm). AR = Argentina, US = United States of America, AU = Australia, PT = Portugal, CA = Canada, CH = Switzerland, DE = Germany, IN = India, FI = Finland, UK = United Kingdom.

**sites did not provide soil detritivore data*

**Site**	**Continent**	**Country**	**Grassland type**	**Start of treatment**	**Elevation**	**Latitude**	**Longitude**	**MAT**	**MAP**	**BLS exposure (in days)**
bari.ar	South America	AR	grassland steppe	2015	786	-41.006	-71.153	8.79	787	20
bldr.us*	North America	US	shortgrass prairie	2008	1633	39.972	-105.234	9.90	487	-
bogong.au	Australia	AU	alpine grassland	2009	1760	-36.874	147.254	5.98	1678	40
burrawan.au	Australia	AU	semiarid grassland	2008	425	-27.735	151.14	18.22	643	40
cbgb.us	North America	US	tallgrass prairie	2009	275	41.785	-93.385	9.26	871	21
cdcr.us	North America	US	tallgrass prairie	2007	270	45.425	-93.212	6.34	740	25
cdpt.us	North America	US	shortgrass prairie	2013	965	41.2	-101.63	9.64	456	17
chilcas.ar	South America	AR	mesic grassland	2013	15	-36.276	-58.266	15.09	955	19
comp.pt	Europe	PT	annual grassland	2012	200	38.829	-8.7914	16.58	564	22
cowi.ca*	North America	CA	old field	2007	50	48.809	-123.63	10.45	762	-
elliot.us	North America	US	annual grassland	2008	200	32.875	-117.052	17.71	344	22
kibber.in*	Asia	IN	alpine grassland	2011	4241	32.3197	78.01	-1.45	400	-

kilp.fi	Europe	FI	tundra grassland	2013	700	69.057	20.875	-3.25	569	-
koffler.ca*	North America	CA	pasture	2010	301	44.024	-79.536	6.28	853	-
konz.us*	North America	US	tallgrass prairie	2007	440	39.071	-96.583	12.08	889	-
lancaster.uk	Europe	UK	mesic grassland	2008	180	53.986	-2.628	8.01	1522	15
marc.ar	South America	AR	grassland	2011	6	-37.715	-57.425	14.32	907	15
mtca.au	Australia	AU	savanna	2008	285	-31.782	117.611	17.75	324	22
ping.au	Australia	AU	old field	2013	338	-32.496	116.973	16.28	456	-
saline.us*	North America	US	mixedgrass prairie	2008	440	39.05	-99.1	12.10	608	-
sgs.us	North America	US	shortgrass prairie	2007	1650	40.817	-104.767	8.95	369	21
smith.us	North America	US	mesic grassland	2007	62	48.207	-122.625	10.18	605	-
spin.us	North America	US	pasture	2007	271.3	38.136	-84.501	12.48	1152	15
ufrec.us*	North America	US	grassland	2013	26	27.433	-81.917	22.43	1284	-
valm.ch	Europe	CH	alpine grassland	2008	2320	46.631	10.3723	0.13	681	15
yarra.au*	Australia	AU	mesic grassland	2014	19	-33.61	150.73	17.32	844	-

Table S2: Piecewise SEM models for each aspect of soil biological activity (i.e., detritivore activity or microbial activity).

**Model 1**	log(plant biomass) ~ NPK x Reduction + logMAP + logH2O + (1|site/block) + (1|year_trt)
**Model 2**	log(plant richness ~ NPK x Reduction+ logMAP + (1|site/block) + (1|year_trt)
**Model 3**	log(soil water content ~ NPK x Reduction + logMAP + (1|site/block) + (1|year_trt)
**Model 4**	log(Soil activity ~ NPK x Reduction + log(plant biomass) + logMAP + log(plant richness) + log(soil water content) + (1|site/block) + (1|year_trt) + (1|days_inserted)

Table S3: Standardized coefficients for each path in the final SEM with detritivore activity as the final response variable.

Response Variable	Explanatory Variable	Std. Estimates	P-Value
Total Plant Biomass (log-scaled)	NPK	0.272	**<0.001**
	Reduction	0.160	**<0.001**
	MAP (log-scaled)	0.454	**0.005**
Plant Species Richness (log-scaled)	NPK	-0.272	**<0.001**
	Reduction	-0.139	**<0.001**
	MAP (log-scaled)	0.117	0.557
Soil Water Content (log-scaled)	NPK	0.033	0.227
	Reduction	0.057	**0.040**
	MAP (log-scaled)	0.453	0.067
	NPK x Reduction	-0.045	0.190
Detritivore Activity (log-scaled)	NPK	0.016	0.719
	Reduction	0.084	**0.031**
	Total Plant Biomass (log-scaled)	-0.092	0.141
	MAP (log-scaled)	0.569	**0.002**
	Plant Species Richness (log-scaled)	0.035	0.634
	Soil Water Content (log-scaled)	0.216	**0.036**

Table S4: The standardized coefficient for each path in the final SEM with microbial activity as the final response variable.

Response Variable	Explanatory Variable	Std. Estimates	P-Value
Total Plant Biomass (log-scaled)	NPK	0.209	**<0.001**
	Reduction	0.092	**0.003**
	Soil Water Content (log-scaled)	-0.010	0.907
	MAP (log-scaled)	0.541	**<0.001**
Plant Species Richness (log-scaled)	NPK	-0.207	**<0.001**
	Reduction	-0.079	**0.003**
	MAP (log-scaled)	-0.077	0.660
Soil Water Content (log-scaled)	NPK	0.034	0.146
	Reduction	0.047	**0.047**
	MAP (log-scaled)	0.500	**0.012**
	NPK x Reduction	-0.048	0.098
Microbial Activity (log-scaled)	NPK	-0.011	0.627
	Reduction	0.008	0.702
	Total Plant Biomass (log-scaled)	-0.096	**0.019**
	MAP (log-scaled)	0.250	**0.043**
	Plant Species Richness (log-scaled)	-0.026	0.579
	Soil Water Content (log-scaled)	0.649	**<0.001**

Table S5: Author contributions.

Name	Question	Analysed data	Contributed to data analysis	Wrote paper	Contributed to writing	Site/data contributor	NutNet Coordinator
Julia Siebert	x	x	x	x	x		
Marie Sünnemann			x	x	x		
Yann Hautier			x		x		
Anita C. Risch	x		x		x	x	
Jonathan D. Bakker					x	x	
Lori Biederman					x	x	
Dana M. Blumenthal					x	x	
Elizabeth T. Borer					x	x	x
Miguel Nuno Bugalho					x	x	
Arthur A.D. Broadbent					x	x	
Maria C. Caldeira					x	x	
Kendi F. Davies					x	x	
Anu Eskelinen					x	x	
Nicole Hagenah					x		
Johannes M.H. Knops					x	x	
Andrew S. MacDougall					x	x	
Rebecca L. McCulley					x	x	
Joslin L. Moore					x	x	
Sally A. Power					x	x	
Jodi N. Price					x	x	

Eric W. Seabloom					x	x	x
Rachel Standish					x	x	
Carly J. Stevens					x	x	
Stephan Zimmermann					x	x	
Nico Eisenhauer	x		x	x	x		

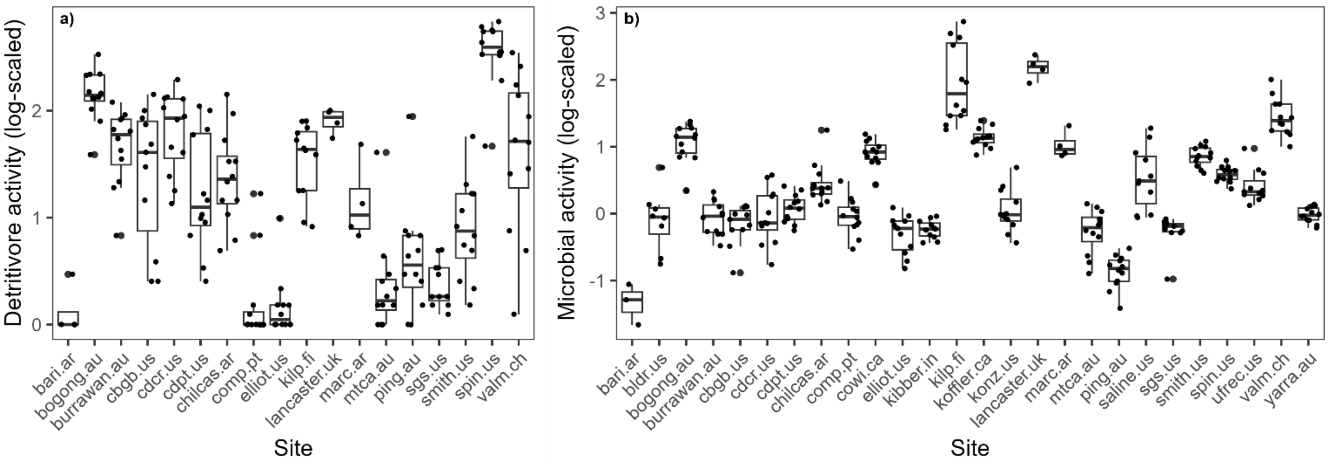

Fig. S1: Variation of a) detritivore activity (log-scaled; data from 18 sites) and b) microbial activity (log-scaled; data from 18 sites) per site.

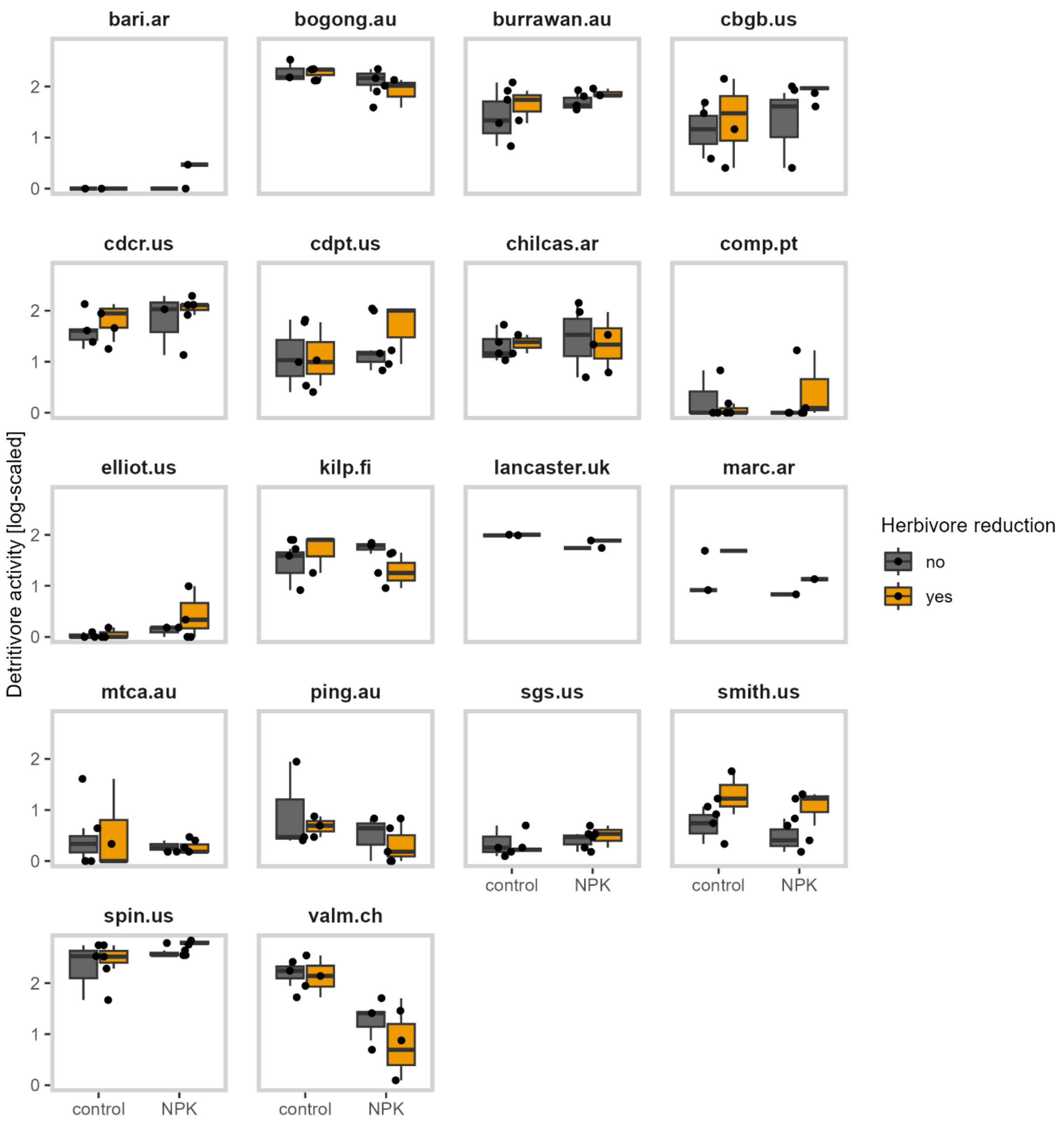

Fig. S2: The effects of NPK fertilization and herbivore reduction on soil detritivore activity (log-scaled) at the 18 sites of the study.

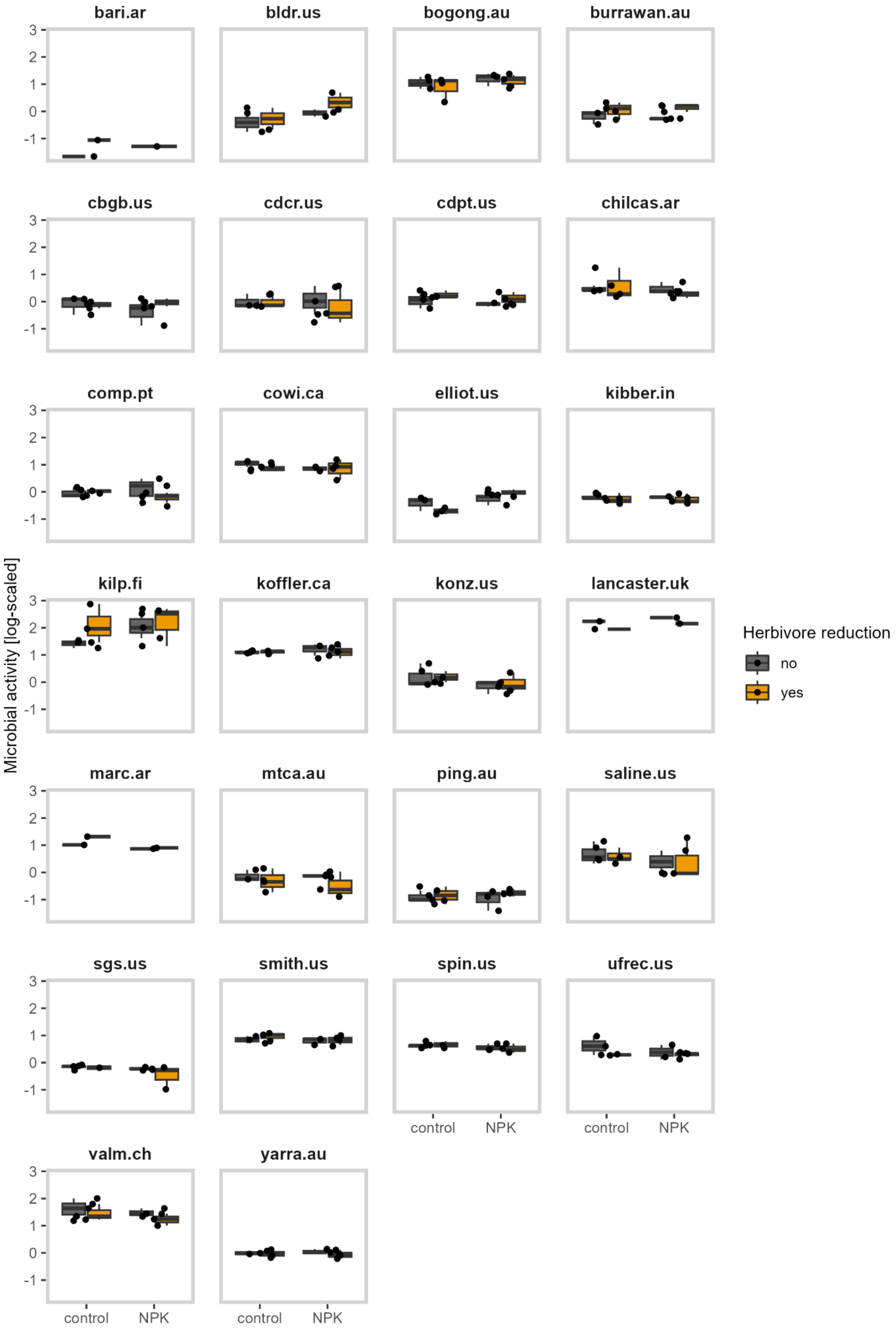

Fig. S3: The effects of NPK fertilization and herbivore reduction on soil microbial activity (log-scaled) at the 26 sites of the study.

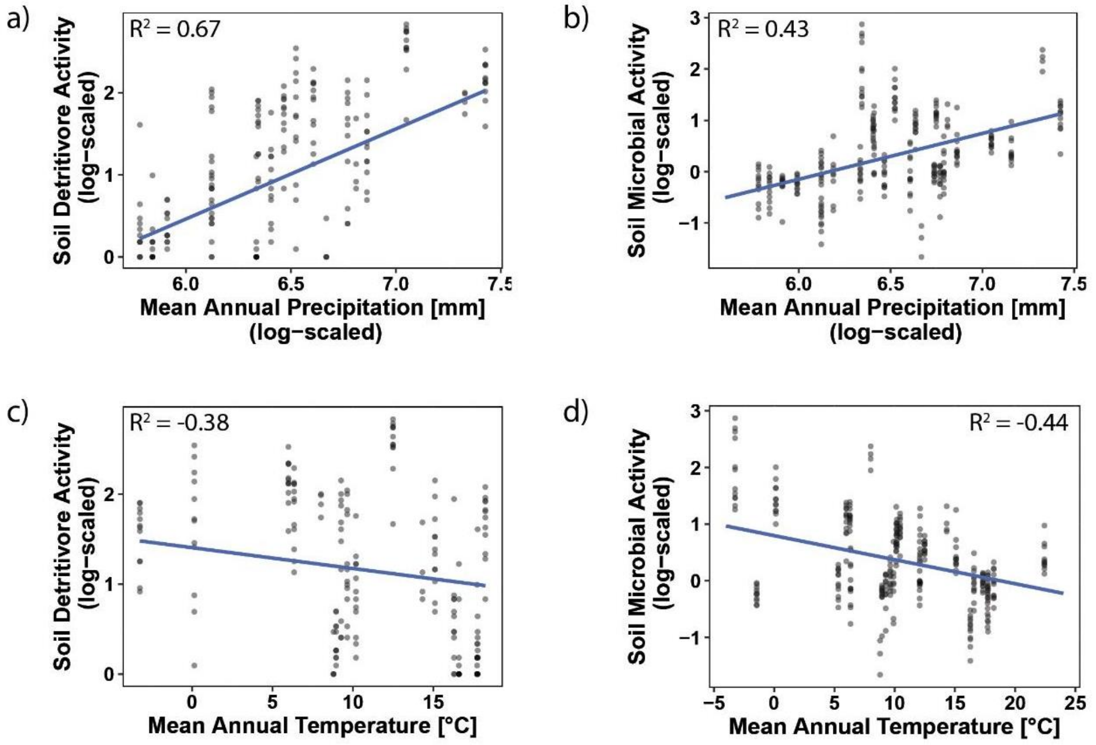

Fig. S4: The effects of MAP on a) detritivore activity (log-scaled; data from 18 sites, p = 0.001) and b) microbial activity (log-scaled; data from 26 sites, p = 0.01); and the effects of MAT on a) detritivore activity (log-scaled; data from 18 sites; p = 0.36) and b) microbial activity (log-scaled; data from 26 sites; p = 0.09). Note differences in scale of axes among graphs. Plots within a site have the same climate and therefore are shown as a vertical stack of points.

31

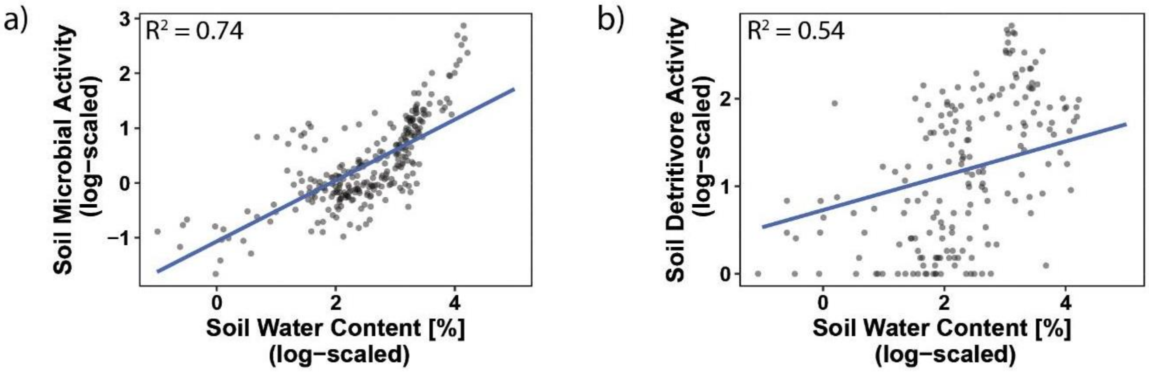

Fig. S5: The relationships between soil water content (log-scaled) and a) microbial activity (log-scaled, $p < 0.001$, 26 sites with 312 plots in total); b) detritivore activity (log-scaled, $p = 0.02$, 18 sites with 216 plots).

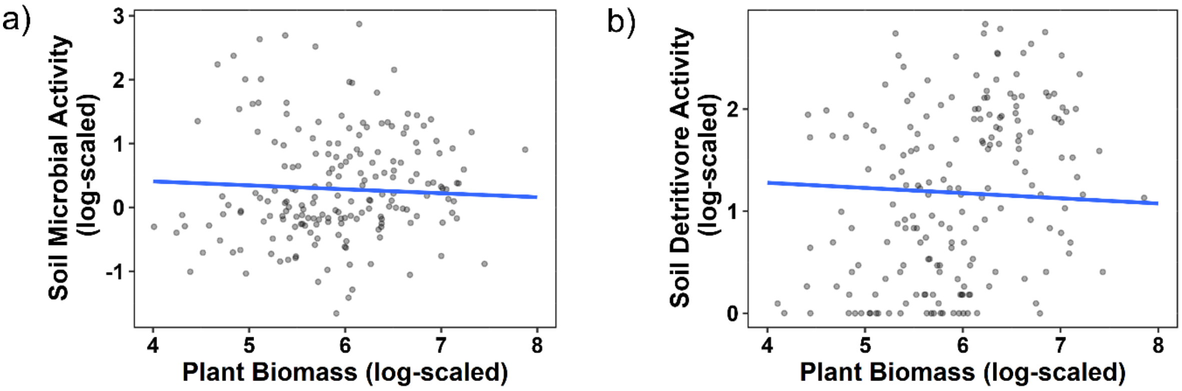

Fig. S6: The relationships between plant biomass (log-scaled) and a) microbial activity (log-scaled, 26 sites with 312 plots in total); b) detritivore activity (log-scaled, 18 sites with 216 plots).

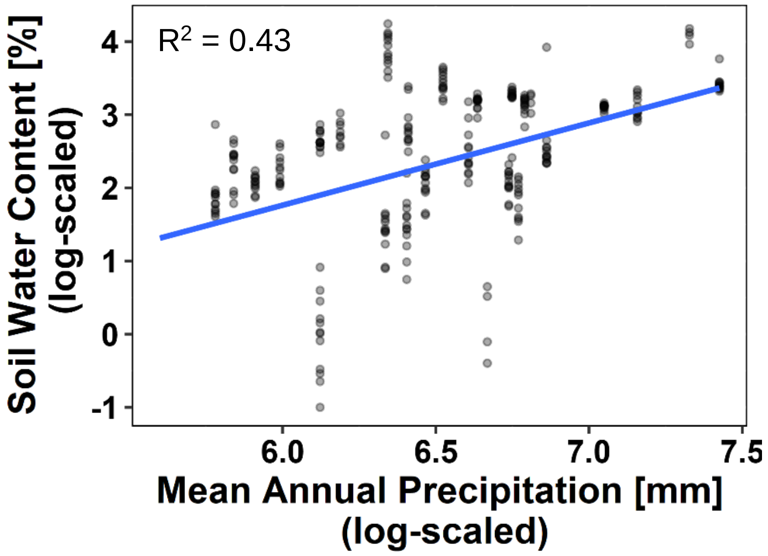

Fig. S7: The relationship between mean annual precipitation (log-scaled) and soil water content (log-scaled, p < 0.001, 26 sites).

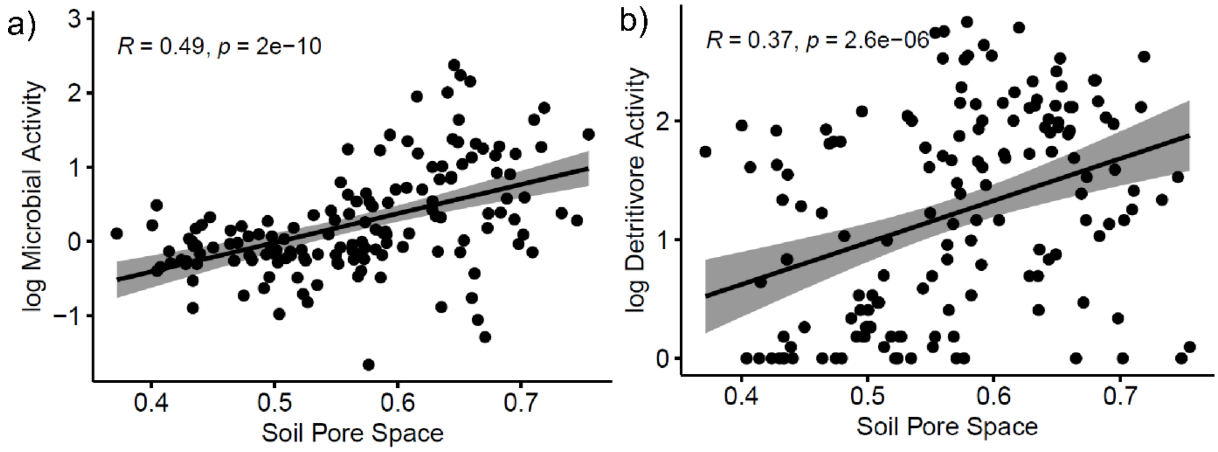

Fig. S8: The relationship between soil pore space and a) microbial activity (log-scaled, p < 0.001, 15 sites); b) detritivore activity (log-scaled, p = 0.02, 15 sites).
