# Supplementary material for: Drivers of soil microbial and detritivore activity across global grasslands
Source: Commun Biol. 2023 Dec 1;6:1220. doi: 10.1038/s42003-023-05607-2 (PMC10692199; doi:10.1038/s42003-023-05607-2)
Supplement: Supplementary file 3 — Description of Supplementary Materials [file 42003_2023_5607_MOESM3_ESM.docx]

**Description of Additional Supplementary Files**

**File name:** Supplementary Data 1

**Description:** The source data of the analyses and graphs on detritivore activity

**File name:** Supplementary Data 2

**Description:** The source data of the analyses and graphs on microbial activity
