# Supplementary material for: Drivers of soil microbial and detritivore activity across global grasslands
Source: Commun Biol. 2023 Dec 1;6:1220. doi: 10.1038/s42003-023-05607-2 (PMC10692199; doi:10.1038/s42003-023-05607-2)
Supplement: Supplementary file 6 — Reporting Summary [file 42003_2023_5607_MOESM6_ESM.pdf]

nature portfolio

Corresponding author(s): Marie Sünnemann

Last updated by author(s): 07.11.2023

Reporting Summary

Nature Portfolio wishes to improve the reproducibility of the work that we publish. This form provides structure for consistency and transparency in reporting. For further information on Nature Portfolio policies, see our Editorial Policies and the Editorial Policy Checklist.

Statistics

For all statistical analyses, confirm that the following items are present in the figure legend, table legend, main text, or Methods section.

n/a	Confirmed	
☐	☒	The exact sample size (*n*) for each experimental group/condition, given as a discrete number and unit of measurement
☐	☒	A statement on whether measurements were taken from distinct samples or whether the same sample was measured repeatedly
☐	☒	The statistical test(s) used AND whether they are one- or two-sided<br>*Only common tests should be described solely by name; describe more complex techniques in the Methods section.*
☐	☒	A description of all covariates tested
☐	☒	A description of any assumptions or corrections, such as tests of normality and adjustment for multiple comparisons
☐	☒	A full description of the statistical parameters including central tendency (e.g. means) or other basic estimates (e.g. regression coefficient) AND variation (e.g. standard deviation) or associated estimates of uncertainty (e.g. confidence intervals)
☐	☒	For null hypothesis testing, the test statistic (e.g. *F*, *t*, *r*) with confidence intervals, effect sizes, degrees of freedom and *P* value noted<br>*Give P values as exact values whenever suitable.*
☒	☐	For Bayesian analysis, information on the choice of priors and Markov chain Monte Carlo settings
☒	☐	For hierarchical and complex designs, identification of the appropriate level for tests and full reporting of outcomes
☒	☐	Estimates of effect sizes (e.g. Cohen's *d*, Pearson's *r*), indicating how they were calculated

Our web collection on statistics for biologists contains articles on many of the points above.

Software and code

Policy information about availability of computer code

Data collection	No software was used for data collection.
Data analysis	We used R to handle and analyse the data.

For manuscripts utilizing custom algorithms or software that are central to the research but not yet described in published literature, software must be made available to editors and reviewers. We strongly encourage code deposition in a community repository (e.g. GitHub). See the Nature Portfolio guidelines for submitting code & software for further information.

Data

Policy information about availability of data

All manuscripts must include a data availability statement. This statement should provide the following information, where applicable:

- Accession codes, unique identifiers, or web links for publicly available datasets
- A description of any restrictions on data availability
- For clinical datasets or third party data, please ensure that the statement adheres to our policy

The data that support the findings of this study are available from the corresponding author upon request.

Research involving human participants, their data, or biological material

Policy information about studies with human participants or human data. See also policy information about sex, gender (identity/presentation), and sexual orientation and race, ethnicity and racism.

Reporting on sex and gender	Does not apply.
Reporting on race, ethnicity, or other socially relevant groupings	Does not apply.
Population characteristics	Does not apply.
Recruitment	Does not apply.
Ethics oversight	Does not apply.

Note that full information on the approval of the study protocol must also be provided in the manuscript.

Field-specific reporting

Please select the one below that is the best fit for your research. If you are not sure, read the appropriate sections before making your selection.

☐ Life sciences ☐ Behavioural & social sciences ☒ Ecological, evolutionary & environmental sciences

For a reference copy of the document with all sections, see nature.com/documents/nr-reporting-summary-flat.pdf

Ecological, evolutionary & environmental sciences study design

All studies must disclose on these points even when the disclosure is negative.

Study description	Field work was conducted in 2015 within the Nutrient Network Global Experiment (www.nutnet.org) at 26 sites. The sites are located in North and South America, Europe, Asia, and Australia and are all dominated by herbaceous or low-statured vegetation. For our study, we sampled plots at each site, which were randomly assigned to one of four: Control, nutrient addition, fenced, nutrient addition and fenced. Each treatment was replicated three times at each site, leading to a total of 12 plots. Fences excluded aboveground herbivores weighing more than 50 g. The plots were 5 x 5 m in size and NPK plots received a fertilization treatment of nitrogen (N), phosphorus (P), and potassium (K). Here, we measured invertebrate feeding activity and microbial activity and used structural equation modeling to
Research sample	A few grams of soil to measure microbial respiration, bait lamina strips to measure feeding activity.
Sampling strategy	Soil was collected from 26 sites six by taking three subsamples per plot (using a soil corer with 5 cm diameter and 12 cm depth), which were then homogenized and sieved using a 2 mm mesh. All soil samples to Anita Risch in Switzerland following a standardized protocol. A subset of these samples was then shipped to a centralised lab at the German Centre for integrative Biodiversity Research in Leipzig, Germany. We ensured sample quality during transit by using postal services with temperature control and fast shipping methods
Data collection	Bait Lamina strips were evaluated by the individual site coordinators, microbial activity was measured in a centralised lab at the German Centre for integrative Biodiversity Research in Leipzig, Germany.
Timing and spatial scale	Samples were taken six weeks before peak plant biomass production (local site coordinators chose specific dates, as seasonality varied across different latitudes).
Data exclusions	Data with NAs were exluded for the SEM models.
Reproducibility	The sampling protocol describes the sampling strategy and analysis in a detailed manner.
Randomization	The NutNet experimental design has plots and treatements randomized. For the measurements of microbial activity, soil samples were randomly attached to the measuring machine.
Blinding	When samples were analysed, researchers did not know about the treatment they had been subjected to before.

Did the study involve field work? ☒ Yes ☐ No

Field work, collection and transport

Field conditions	Sampling took always place 6 weeks before peak biomass, so always in summer.
Location	As its many different sites, these information can be found in the supplements of the manuscript.
Access & import/export	By car and then foot, always in compliance with local restrictions.
Disturbance	Does not apply.

Reporting for specific materials, systems and methods

We require information from authors about some types of materials, experimental systems and methods used in many studies. Here, indicate whether each material, system or method listed is relevant to your study. If you are not sure if a list item applies to your research, read the appropriate section before selecting a response.

Materials & experimental systems

n/a	Involved in the study	
☒	☐	Antibodies
☒	☐	Eukaryotic cell lines
☒	☐	Palaeontology and archaeology
☒	☐	Animals and other organisms
☒	☐	Clinical data
☒	☐	Dual use research of concern
☐	☒	Plants

Methods

n/a	Involved in the study	
☒	☐	ChIP-seq
☒	☐	Flow cytometry
☒	☐	MRI-based neuroimaging

Dual use research of concern

Policy information about dual use research of concern

Hazards

Could the accidental, deliberate or reckless misuse of agents or technologies generated in the work, or the application of information presented in the manuscript, pose a threat to:

No	Yes	
☒	☐	Public health
☒	☐	National security
☒	☐	Crops and/or livestock
☒	☐	Ecosystems
☒	☐	Any other significant area

Experiments of concern

Does the work involve any of these experiments of concern:

No	Yes	
☒	☐	Demonstrate how to render a vaccine ineffective
☒	☐	Confer resistance to therapeutically useful antibiotics or antiviral agents
☒	☐	Enhance the virulence of a pathogen or render a nonpathogen virulent
☒	☐	Increase transmissibility of a pathogen
☒	☐	Alter the host range of a pathogen
☒	☐	Enable evasion of diagnostic/detection modalities
☒	☐	Enable the weaponization of a biological agent or toxin
☒	☐	Any other potentially harmful combination of experiments and agents
